# Suicide attempt and completed suicide in adolescents and young people on from the social health determinants: A systematic review 

**DOI:** 10.15649/cuidarte.4184

**Published:** 2025-04-29

**Authors:** Ladini Sunanda Hernández Bello, Andrés Mauricio Ríos Paternina, Fernando de la Hoz Restrepo

**Affiliations:** 1 Docente Asistente, Universidad de Cartagena, Cartagena, Colombia. Grupo Cuidado a la Salud de los Colectivos. PhD(c) Salud Pública, Universidad Nacional de Colombia. lhernandezb2@unicartagena.edu.co Universidad de Cartagena Universidad de Cartagena Cartagena Colombia lhernandezb2@unicartagena.edu.co; 2 Médico, Especialista en Auditoria en Salud. Docente Corporación Universitaria Rafael Núñez. Cartagena, Colombia. andreu1260@gmail.com Corporación Universitaria Rafael Núñez Corporación Universitaria Rafael Núñez Cartagena Colombia andreu1260@gmail.com; 3 Docente Titular, Universidad Nacional de Colombia, Bogotá, Colombia. Grupo Epidemiologia y Evaluación en Salud. fpdelahozr@unal.edu.co Universidad Nacional de Colombia Universidad Nacional de Colombia Bogotá Colombia fpdelahozr@unal.edu.co

**Keywords:** Social Determinants of Health, Suicide, Suicide Attempt, Adolescent, Determinantes Sociales de la Salud, Suicidio, Intento de Suicidio, Adolescente, Determinantes Sociais da Saúde, Suicídio, Tentativa de Suicídio, Adolescente

## Abstract

**Introduction:**

Suicidal behavior is an important health problem, frequently studied from a risk perspective. Evidence that transcends this hegemonic view is required.

**Objective:**

To identify the structural and intermediate social health determinants associated with attempted suicide and completed suicide in Latin American adolescents and youth, according to published literature.

**Materials and Methods:**

Systematic review following PRISMA recommendations, performed in LILACS, Google academic and Pubmed using keywords. Primary ecological studies performed in Latin America were included, which were evaluated for confounding bias, data quality and ecological fallacy.

**Results:**

Initially, 23,770 documents were located, and 10 were finally included. The structural determinants associated with suicide include being male, aged 15-24 years, having a high Gini index, having a low Gross Domestic Product (GDP) per capita, and being Catholic or Evangelical. While the suicide attempt was due to educational backwardness, being a woman, and living in the municipal seat. The intermediate determinants for suicide attempts were tobacco and alcohol consumption, violent episodes, and depression.

**Discussion:**

The proposed theoretical model offers a novel view of the problem, moving away from individual responsibility and giving an active role to lifestyle-related conditions contributing to health inequities.

**Conclusion:**

Social determinants offer a novel view for developing new prevention actions; however, empirical evidence from Latin America remains contradictory, and half of the studies reviewed were affected by confounding bias. Therefore, these associations should be interpreted with caution.

## Resumen

**Introducción:**

La conducta suicida es un importante problema de salud, estudiada con frecuencia desde el enfoque de riesgo. Se requiere evidencia que trascienda esta visión hegemónica.

**Objetivo:**

Identificar los determinantes sociales de la salud estructurales e intermedios asociados al intento de suicidio y suicidio en adolescentes y jóvenes latinoamericanos de acuerdo con la literatura publicada.

**Materiales y métodos:**

Revisión sistemática siguiendo las recomendaciones PRISMA realizadas en LILACS, Google académico y Pubmed mediante el uso de palabras clave. Se incluyeron estudios primarios ecológicos realizados en Latinoamérica, a los que se les evaluó el sesgo de confusión, calidad del dato y falacia ecológica.

**Resultados:**

Inicialmente se localizaron 23 770 documentos, se incluyeron finalmente 10. Los determinantes estructurales que se asociaron al suicidio fue ser hombre, edad entre 15-24 años, índice Gini, bajo Producto Interno Bruto per cápita, ser católico o evangélico. Mientras que para el intento de suicidio fueron el rezago educativo, ser mujer y vivir en cabecera municipal. Los determinantes intermedios para el intento de suicidio fueron consumo de tabaco y alcohol, episodios violentos y depresión.

**Discusión:**

El modelo teórico propuesto, ofrece una visión novedosa del problema, se aleja de la responsabilidad individual y confiere participación activa a condiciones relacionadas con los modos de vida que generan inequidades en salud.

**Conclusiones:**

Los determinantes sociales ofrecen una visión novedosa con miras hacia nuevas acciones de prevención, sin embargo, la evidencia empírica producida en Latinoamérica es contradictoria y la mitad de los estudios incurrieron en sesgo de confusión, por ello las asociaciones deben ser tomadas con precaución.

## Resumo

**Introdução:**

O comportamento suicida é um importante problema de saúde, frequentemente estudado sob uma perspectiva de risco. São necessárias evidências que transcendam essa visão hegemônica.

**Objetivo:**

Identificar os determinantes sociais estruturais e intermediários da saúde associados às tentativas de suicídio e ao suicídio em adolescentes e jovens latino-americanos de acordo com a literatura publicada.

**Materiais e métodos:**

Revisão sistemática seguindo as recomendações PRISMA realizada nas bases de dados LILACS, Google Acadêmico e Pubmed utilizando palavras-chave. Foram incluídos estudos ecológicos primários conduzidos na América Latina, e o viés de confusão, a qualidade dos dados e a falácia ecológica foram avaliados.

**Resultados:**

Inicialmente, foram localizados 23.770 documentos, e finalmente incluídos 10. Os determinantes estruturais que foram associados ao suicídio foram ser do sexo masculino, ter idade entre 15-24 anos, índice de Gini, baixo Produto Interno Bruto per capita e ser católico ou evangélico. Já para a tentativa de suicídio os fatores mais prováveis foram atraso educacional, ser mulher e residir em capital municipal. Os determinantes intermediários para tentativa de suicídio foram uso de tabaco e álcool, episódios violentos e depressão.

**Discussão:**

O modelo teórico proposto oferece uma nova visão do problema, afasta-se da responsabilidade individual e confere participação ativa às condições relacionadas aos estilos de vida que geram iniquidades em saúde.

**Conclusões:**

Os determinantes sociais oferecem uma nova visão com vistas a novas ações de prevenção, entretanto, as evidências empíricas produzidas na América Latina são contraditórias e metade dos estudos incorrem em viés de confusão, portanto as associações devem ser vistas com cautela.

## Introduction

Suicide attempts and completed suicides are part of a process known as suicidal behavior, a complex and multifactorial phenomenon whose hegemonic causal explanation stems from psychopathology, which views these events as occurring within the course of a mental disorder^1^,^2^. According to the World Health Organization (WHO), there are 800,000 deaths by suicide each year, with the vast majority involving a mental or behavioral disorder or psychoactive substance use. The estimated rate is 11.4 deaths per 100,000 inhabitants^3^. 

Although deaths by suicide are currently considered a public health problem, the situation with suicide attempts is even more alarming when you consider that for every person who dies by suicide, 20 others have already attempted it^4^. A previous suicide attempt is the main individual risk factor for suicide, which is defined as the deliberate act of taking one's own life. In contrast, attempted suicide refers to multiple deliberate and self-destructive behaviors initiated by an individual with the intent to cause serious harm or end their life, but that, due to some circumstances, does not result in a fatal outcome^5^. 

Suicide is the second leading cause of death among youth aged 15-29 years, with suicide attempts also being more frequent in this population after traffic accidents^5^. In this sense, due to their development stage, adolescents are in the middle of a series of changes, marked by increased conflicts with their parents, distancing from home norms and values, exploration of sexuality, rupture with the usual group of friends, and mood fluctuations. These changes can contribute to engaging in risky behaviors, influenced by multiple social determinants that make suicide attempts persist into adulthood^6^. Additionally, youth is a crucial stage for achieving life autonomy as individuals transition from school to the workforce, shape their identity, and navigate dreams and aspirations in different aspects of life. Therefore, this stage is a crucial moment of change in people's lives^6^. 

Hence, there is an interest in studying the social determinants of health and their influence on this phenomenon. Research from this perspective in Latin America remains limited compared to research using a risk-based approach, which has extensively examined biological, psychological, familial, and social factors associated with suicidal behavior in adolescents^7^. Furthermore, methodologically, the ecological design is the predominant design for studying the phenomenon using the model of the social determinants of health, particularly in European and Anglo-Saxon settings. This methodological trend suggests that studies following this approach probably correspond to the same design. Likewise, a Google Scholar search for systematic reviews on the phenomenon using the WHO model of social determinants of health in Latin America yielded no results. 

A review of the published evidence on the relationship between social determinants of health and suicide attempts or completed suicides among adolescents and youth is considered pertinent. In this way, it is possible to contribute to an understanding of the phenomenon from a social perspective, moving beyond the psychopathological view, which often overlooks the economic, political, and social realities that adolescents and youth in Latin American countries live in. Accordingly, this research aimed to identify the structural and intermediate social determinants of health associated with suicide attempts and completed suicides among adolescents and youth in Latin America, according to published literature. 

## Materials and Methods

This secondary research study follows a systematic review methodology in accordance with the Preferred Reporting Items for Systematic Reviews and Meta-Analyses (PRISMA) statement^8^. The LILACS, Google Scholar, and PubMed databases were systematically consulted in November 2023. The search was restricted to studies conducted in Latin America over the past 10 years, aiming to know the state of evidence on the social determinants of health associated with suicide and suicide attempts in the region. Additionally, scientific literature published in indexed journals builds on previous knowledge, which may become outdated due to the rapid growth of research and publication. 

The central theme of the studies had to focus on the social determinants of health associated with suicide attempts or completed suicides among adolescents and/or youth. The keywords "socioeconomic factors," "social determinants of health," "suicide," "suicide attempt," and "adolescent" were used and consulted in the DeCS-MeSH Health Sciences Descriptors. Search strings were constructed in Spanish, English, and Portuguese using the Boolean operators AND and OR, as follows:

- Socioeconomic Factors AND suicide OR suicide Attempted AND adolescent OR youths

- Factores Socioeconómicos AND suicidio OR intento de suicidio AND adolescente OR jóvenes

- Fatores Socioeconômicos AND Suicídio OR Tentativa de Suicídio AND Adolescente OR jovens

- Social Determinants of Health AND suicide OR suicide Attempted AND adolescent OR youths

- Determinantes Sociales de la Salud AND suicidio OR intento de suicidio AND adolescente OR jovenes

- Determinantes Sociais da Saúde AND Suicídio OR Tentativa de Suicidio AND Adolescente OR jóvenes

**Inclusion and exclusion criteria.** Primary ecological studies were included, provided they focused on social determinants of health associated with attempted or completed suicides among adolescents and/or youth in Latin America. Studies were excluded if full-text access was unavailable and payment was required for viewing or downloading. Additionally, gray literature was excluded as it often consists of lengthy documents and lacks peer review. Narrative reviews and opinion articles were also excluded. 

**Study selection.** To select the studies, first, each reviewer screened article titles. If a title aligned with the research topic, the abstract was read. Studies that met the inclusion and exclusion criteria were then downloaded and stored using the Mendeley reference manager, with each reviewer maintaining a designated folder. Following the individual review process, two meetings were held, during which the three reviewers jointly examined the folders and saved articles. A folder was created for four disputed articles from the initial selection. Each article was then reassigned to two reviewers who had not previously assessed it in the initial review. Based on the established inclusion and exclusion criteria, they made the final selection decision. 

**Risk of bias assessment.** As there are no tools for assessing bias or conducting a critical appraisal of ecological studies to our knowledge, we analyzed the biases present in the articles included in this review based on criteria described in the literature^9^,^10^. These criteria identify three biases which ecological studies are susceptible to: confounding bias, data quality bias, and ecological fallacy. For this purpose, Morgenstern's recommendations^11^ were followed, as outlined below:

- Confounding bias: We checked whether the ecological variables in the included studies were treated as covariates to obtain an adjusted estimate of the effect —in this case, the outcomes of suicide attempts and completed suicides. Another possibility was that the researchers of the studies obtained standardized rates for these variables. If the ecological study involved comparing groups from multiple geographic areas, we checked whether the authors stratified the data to account for regions with less geographic variation in both exposure and rates^9^,^10^.

- Data quality bias: We checked whether the authors used regression models to estimate observation units that lacked data and whether cases were re-categorized^10^,^11^.

- Ecological fallacy: We examined whether the authors used ecological regression rather than correlation to estimate the magnitude of the association between suicide attempts and/or completed suicides and social determinants of health. We also reviewed whether the studies used data aggregated at the smallest possible geographic units of analysis. Additionally, we examined whether the studies avoided extrapolating results to the individual level and instead focused only on conclusions that apply strictly to the population level^11^.

**Variables and data extraction.** Three types of variables were extracted: a) study characteristics (authors, year of publication, country, language, study design, participants), b) social determinants associated with suicide attempts, and c) social determinants associated with completed suicide, including statistical measures (e.g., Pearson's correlation, Spearman's correlation, Odds Ratio). Data was extracted using a Microsoft Excel workbook created by the reviewers. This facilitated duplicate identification and removal while also enabling comparisons of the quality of the extracted data across studies.

**Data analysis.** A qualitative analysis of the data was conducted, synthesizing the general characteristics of the studies and the social determinants of health through summaries and descriptive tables.

**Ethical considerations.** As this study is secondary research, it complies with Colombian Law 23 of 1983 on copyright, which mandates citation of authors' names, pseudonyms, and the titles of original works used in this review. 

 The full dataset is freely accessible for public consultation on Mendeley Data^12^.

## Results

**Figure 1:**
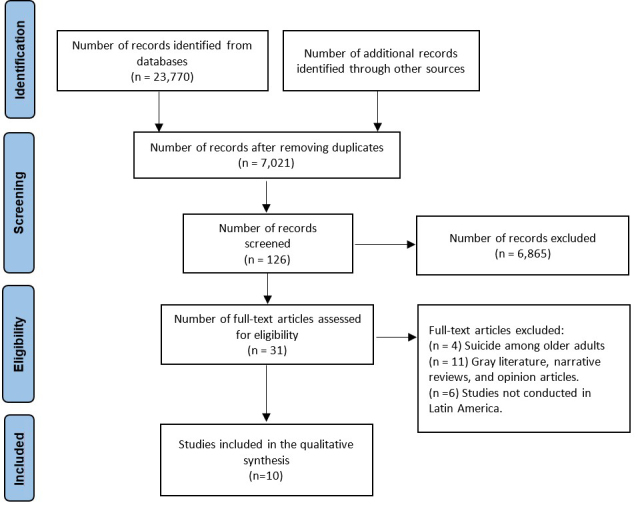
PRISMA flow diagram

A total of 23,770 documents were identified in the initial search. After removing duplicates, 7,021 records remained, of which 6,895 were excluded for not meeting the research objectives. This left 126 records, from which 31 studies that met the research objectives were selected. Finally, 10 studies that met the inclusion criteria were included in the review (see Figure 1). All included studies followed an ecological design. Three studies were conducted in Brazil^13^-^15^, three in Mexico^16^-^18^, three in Colombia^19^-^21^, and one in Ecuador^22^. Additionally, 60% (6)^14^,^17^,^19^ of the studies were published within the last five years (Figure 1). 

**Suicide attempt and completed suicide**

The studies reported varying relative frequencies and rates of suicide attempts and completed suicides, depending on the population studied. Only three studies focused on attempted suicide^16^,^18^,^19^. Among 4,855 deaths by suicide in adolescents and youth (10-24 years), Gerstner^22^ reported a suicide rate of 10.5 per 100,000 in 2007 (13.4 in males and 7.7 in females); this rate had declined to 9.6 by 2012 (12.5 in males and 6.7 in females). 

Among adolescents aged 10-19 years, Jaén^13^ reported a suicide rate of 2.34 per 100,000 in 2006, which increased to 2.64 per 100,000 in 2015. In the same age group, Luna^16^ reported a suicide attempt prevalence of 2.74%, with 1.45% of cases occurring within 12 months prior to the survey. Similarly, Rivera^17^ reported a lifetime suicide attempt rate of 3.9%, while it was 1.8% within the past 12 months. 

Borges^14^ analyzed all completed suicides in Brazil between 2000 and 2011 and found a suicide rate of 10.47 in men and 2.45 in women in 2000. This rate had increased to 12.48 per 100,000 inhabitants in men and 2.42 per 100,000 inhabitants in women by 2011. The remaining studies did not report prevalence rates for attempted or completed suicide cases, as their objective was to analyze the association between social determinants and the total number of attempted and completed suicide cases recorded in government databases. These results are presented in Table 1 for further detail. 

**Table 1:** Summary of studies included in the review

Lead author, year	Participants	Associated structural determinants	Associated intermediate determinants
Gerstner, 2018^22^	4855 completed suicide cases in adolescents and youth.	Male r= 1.9 (95% CI: 1.7-2.1) Ages between 15-24 r=2.9 (95% CI: 2.4-3.3)	Not reported
Jaén 2019^13^	Completed suicide in adolescents	High Gini index (inequality) β= 9.63 (CI 95%: 2.31-16.96) Low GDP per capita β= -5.82 (CI 95%:-8.41-3.23)	High unemployment rate β= 0.06 (CI 95%: 0.02 -0.10)
Dávila 2018^16^	21,509 adolescents ages 10-19 with suicide attempts	Being female B= 9.6 (CI 95%: 5.3-17.5) Educational backwardness B= 1.6 (CI 95%: 1.1-2.3)	Tobacco use B= 2.4 (CI 95%: 1.5-3.8) Alcohol B= 4.1 (CI 95%: 2.6-6.2) Episode of violence B= 5.8 (IC: 2.7-12.4)
Rivera 2020^17^	17,925 adolescents ages 10-19 with suicide attempts	Not reported	Tobacco consumption RM=2.09 (CI 95%: 1.42-3.07) Alcohol consumption RM=2.32 (CI 95%: 1.77-3.03) Depressive symptoms RM= 6.47 (CI 95%: 4.91-8.51) History of sexual abuse RM= 6.76 (CI 95%: 4.60-9.96)
Murillo 2022^19^	32 076 cases of attempted suicide among adolescents.	Being male OR= 0.29 (CI95%: 0.28-0.29) Living in a municipal seat OR= 1.42 (CI95%: 1.38-1.46)	Not reported
Campo, 2015^21^	Completed suicide cases	Gini index (inequality) r= 0.70; p < 0.001	Not reported
Campo, 2014^20^	Completed suicide cases	Gini index (inequality) r=-0.401; p=0.052	Not reported
Manríquez 2015^18^	Completed suicide among adolescents	Income, suicide prevention spending, GDP stagnation, sex, and age. R2 0.6989	Unemployment, weekly working hours, access to health institutions. R2 0.6989
Borges D, 2015 Brazil^14^	Completed suicide among adolescents	Gini index RR= 1.05 (CI95%: 1.01-1.09) Per capita income RR= 0.97 (CI95%: 0.95-0.99) No basic education RR= 1.01 (IC95%: 1.01-1.02)Catholics RR= 1.02 (CI95%: 1.01-1.03)Evangelicals RR= 1.02 (CI95%: 1.01-1.02)	Average number of residents per household RR= 0.32 (CI95%: 0.29-0.35) Divorced RR= 0.96 (CI95%: 0.93-0.98) Urbanization rate RR= 0.99 (CI95%: 0.98-0.99)
Dantas A, 2018 Brazil^15^	Completed suicide	Income ratio between the richest 10% and the poorest 40% (Moran index 0.44526 p≤0.001). Gini index (Moran index 0.46629 p≤0.001).	Unemployment rate among those ≥18 years old (Moran index 0.51899 p≤0.001). Percentage of population living in dense households (Moran index 0.85111 p≤0.001).

Note: r (Pearson correlation coefficient), β (beta), B (logistic regression), OR (odds ratio), R2 (R-squared), RR (relative risk), MR (odds ratio) .

**Social determinants of health according to the WHO model**

The WHO model of social determinants of health establishes determinants as factors or characteristics that influence individuals' health. These determinants act or interact at different organizational levels, ultimately determining the health status of a population^23^. 

According to the WHO model, determinants are classified as structural and intermediate. The former refers to social position and contexts that generate social stratification, including factors such as income, education, occupation, social class, gender, race/ethnicity, and macroeconomic and social policies. The latter includes the exposures and vulnerabilities faced by population groups within various social levels, such as the family, school, or workplace where individuals live and develop^24^. 

Based on the WHO model, the social determinants reported by the studies included in the review were recategorized into structural and intermediate determinants. The structural determinants associated with completed suicide among youth included being male, aged between 15-24 years, having a Gini index close to one (high inequality), having low GDP per capita^13^,^18^,^22^, and being Catholic or Evangelical^14^. However, the evidence may be contradictory, as two studies reported that completed suicide rates were independent of the inequality index, suggesting that no relationship exists between suicide and this determinant^20^,^21^. 

For suicide attempts, the structural determinants identified were educational backwardness, being female^16^, and living in a municipal capital^19^. Regarding intermediate determinants of suicide attempts, tobacco use, episodes of violence^16^,^17^, alcohol consumption, and depressive symptoms were identified^17^ (Table 2). 

**Table 2:** Assessment of the risk of bias in the studies reviewed

Lead author, year	Confusion bias	Data quality bias	Ecological fallacy
Gerstner, 2018^22^	Absent	Not reported	Absent
Jaén 2019^13^	Present	Present	Absent
Dávila 2018^16^	Present	Absent	Absent
Rivera 2020^17^	Present	Absent	Absent
Murillo 2022^19^	Absent	Absent	Absent
Campo, 2015^21^	Present	Present	Present
Campo, 2014^20^	Present	Present	Present
Manríquez 2015^18^	Absent	Not reported	Absent
Borges, 2015^14^	Absent	Absent	Absent
Dantas, 2018^15^	Absent	Not reported	Absent

## Discussion

The results of this review can be interpreted in several ways. First, research on the social determinants of health in relation to suicide attempts and completed suicide is scarce in Latin America. The research that was found corresponded to ecological studies. This lack of research constitutes an opportunity for generating new knowledge. Understanding suicide attempts and completed suicide from a different perspective beyond the traditional psychopathological and biomedical approach is necessary, as this approach fails to establish a causality for the phenomenon. 

Secondly, the WHO's theoretical model of social determinants of health offers a novel perspective of the problem. By shifting the focus away from individual responsibility, this model confers active participation to the cumulative impact of populations' lifestyle-related conditions that drive health inequities, ultimately determining the health status of population groups^25^,^26^. Although ecological studies—which dominate research on determinants— cannot establish direct causality, their strength lies in their approach to a population-level and collective explanation of the problem, far removed from the individualistic perspectives often emphasized in other research designs. 

Thirdly, research adopting this theoretical stance does not go deeper into the WHO model, as it tends to analyze a few structural or intermediate determinants. In this review, the most frequently studied structural determinants include inequality index, per capita income, and sex, which have been associated with completed suicide rates^13^-^19^. However, the evidence is contradictory, as two studies reported no significant relationship between suicide rates and these determinants^20^,^21^. According to Naranjo^27^, further studies are needed to analyze the relationship between inequality and suicide in Latin America and to clarify the causal association between economic variables and deaths by suicide. 

As shown in the results of this research, there are currently few studies that suggest a relationship between suicide and inequality. Brazil, Mexico, and Colombia have demonstrated interest in this problem, but research has failed to examine in depth how inequality influences suicide rates. According to Durkheim^28^, suicide attempts and suicide rates tend to increase with economic imbalance, as social cohesion weakens, affecting individual's perceptions and cognitions, which may lead to this outcome. However, empirical evidence is needed to support this position in this region. 

In other regions of the world, more empirical data on this problem exists. Chandler^29^ analyzed suicide rates in the United Kingdom, reporting that socioeconomic inequalities lead to adverse experiences and emotions, which, in turn, lead to suicide. Curtis^30^, in New Zealand, concluded that income inequality has contributed to the increase in suicide rates. Harper^31^, in the USA, found that economic recessions of different periods, such as those in 2000 and 2010, increased suicide mortality by 0.14 deaths per 100,000 population. 

According to the studies in this review, intermediate determinants such as tobacco and alcohol use, episodes of violence, and depressive symptoms were associated with suicide attempts. Naranjo^27^ affirms that individuals —particularly women— exposed to situations of violence, such as sexual abuse, are 12 to 20 times more likely to attempt suicide due to the emotional impact and psychological suffering caused by situations of violence on the individual^32^. 

Several studies^33^-^35^ affirm that youth and adolescents who use tobacco and alcohol may experience depressive symptoms, increasing their risk of suicide attempts and completed suicides. A review by Hernandez^7^ found that, in adolescents, the risk of suicide increased with psychoactive substance use and the presence of depression. Given this evidence, examining how structural and intermediate determinants interact in attempted and completed suicide outcomes among adolescents and youth. This is particularly critical, as suicide prevention during this stage of life is essential to protecting their productive potential^36^. 

This article provides a synthesized overview of research on suicide and suicide attempts in Latin America from the perspective of the WHO model of social determinants of health. It also offers a different and novel perspective of causal associations between inequality, per capita income, sex, and tobacco and alcohol consumption, which are structural and intermediate determinants of the problem. These results suggest that attempted and completed suicide prevention actions should go beyond promoting healthy lifestyles for mental health and early detection of mental disorders. Political and governmental willingness is required to reduce health inequities and implement comprehensive, integrated actions that influence people's lifestyles and, consequently, their health^37^. 

At the same time, it is important to point out some limitations related to the quality of the reviewed studies. The first limitation concerns the inherent weaknesses of ecological studies, which, as observed, frequently present confounding bias. This can lead researchers to establish false associations. Therefore, it is necessary to design ecological studies that manage to control this bias by incorporating standardized outcome rates and utilizing regional stratification when comparing groups with less geographic variability^37^. 

Another bias observed in the reviewed studies is data quality bias. While this issue is beyond the researchers' control, it requires data cleaning, categorization, and tabulating data again to improve the quality of the information.

A limitation of this review is the exclusion of gray literature, which may have introduced selection bias. This bias may have reduced the likelihood of locating a broader body of research on the problem, particularly studies aligned with the theoretical model under consideration. Likewise, due to the heterogeneity in the measurement of both outcome and independent variables, conducting meta-analyses was not possible. For future reviews, analyzing more homogeneous studies that would enable quantitative synthesis is recommended, thereby strengthening the contributions to knowledge of this problem. 

## Conclusions

In this review, the association of social determinants with the phenomenon under study was found contradictory. Some studies reported that the higher the inequality, the higher the suicide rate, while others concluded that suicide rates do not depend on inequality level. Per capita income was another structural determinant. Some studies suggested that low GDP increases suicide rates, while others indicated that low GDP acts as a protective factor. In addition, death by suicide was higher in men, whereas suicide attempts were higher among women. 

The intermediate determinants associated with suicide attempts included alcohol and tobacco use, symptoms of depression, unemployment, and a history of sexual or other forms of violence. Finally, half of the studies reviewed incurred confounding bias, indicating that these associations should be interpreted with caution.

## References

[1] 1. Stauffacher M, Stiefel F, Dorogi Y, Michaud L. Observational study of suicide in Switzerland: comparison between psychiatric in- and outpatients. *Swiss Med Wkly.* 2022;152:w30140. 10.4414/smw.2022.w30140 35230043

[2] 2. Van Veen M, Wierdsma AI, van Boeijen C, Dekker J, Zoeteman J, Koekkoek B et al. Suicide risk, personality disorder and hospital admission after assessment by psychiatric emergency services. *BMC Psychiatry.* 2019;19(1):157. 10.1186/s12888-019-2145-0 PMC6533743 31122268

[3] 3. Organización Panamericana de la Salud. Prevención del suicidio, 2019. Consulta: Octubre 2, 2024. Disponible en: https://www.who.int/es/news/item/17-06-2021-one-in-100-deaths-is-by-suicide

[4] 4. Organización Mundial de la Salud. Una de cada 100 muertes es por suicidio. 2021. Consulta: Octubre 2, 2024. Disponible en: https://www.who.int/es/news/item/17-06-2021-one-in-100-deaths-is-by-suicide

[5] 5. Parra-Uribe I, Blasco-Fontecilla H, Garcia-Páres G, Martínez-Naval L, Valero-Coppin O, Cebrià-Meca A et al. Risk of re-attempts and suicide death after a suicide attempt: A survival analysis. *BMC Psychiatry.* 2017;17(1):163. 10.1186/s12888-017-1317-z PMC5415954 28472923

[6] 6. Cacho Becerra Z, Silva Balarezo M, Yengle Ruíz C. El desarrollo de habilidades sociales como vía de prevención y reducción de conductas de riesgo en la adolescencia. *Transformación* 2019;15(2):186-205. http://scielo.sld.cu/scielo.php?pid=S2077-29552019000200186&script=sci_arttext

[7] 7. Hernández-Bello L, Hueso-Montoro C, Gómez-Urquiza JL, Cogollo-Milanés Z. Prevalencia y factores asociados a la ideación e intento de suicidio en adolescentes: revisión sistemática.* Rev Esp Salud Pública.* 2020;94:e202009094. https://digibug.ugr.es/handle/10481/63924 PMC11583067 32909551

[8] 8. Urrutia G, Bonfill X. Declaracion PRISMA: una propuesta para mejorar la publicación de revisiones sistemáticas y metaanálisis. *Med clín.* 2010;135(11):507-511. 10.1016/j.medcli.2010.01.015 20206945

[9] 9. Blanco-Becerra LC, Pinzón-Flórez CE, Idrovo AJ. Estudios ecológicos en salud ambiental: más allá de la epidemiología. *Biomédica.* 2015;35(2):191-206. 10.7705/biomedica.v35i0.2819 26535754

[10] 10. López-Abente G. Estudios ecológicos. En: Royo M, Damian J. Método epidemiológico. Escuela Nacional de Sanidad (ENS), Madrid: ENS - Instituto de Salud Carlos III. 2009. p: 140-148. Citado: 02/12/2024 Disponible en: https://sb86eb09335ad47f5.jimcontent.com/download/version/1326191515/module/3699358852/name/ENS%202009%20-Manual%20de%20Metodolog%C3%ADa%20Epidemiol%C3%B3gica%20Ministerio%20Sanidad-.pdf

[11] 11. Morgenstern H. Uses of Ecologic Analysis in Epidemiologic Research. *American journal of public health*. 1982;72(12):1336-1344. https://ajph.aphapublications.org/doi/abs/10.2105/AJPH.72.12.1336 10.2105/ajph.72.12.1336 PMC1650553 7137430

[12] 12. Hernández Bello L, Ríos Paternina A, De la Hoz Restrepo F. Intento de suicidio y suicidio consumado desde los determinantes sociales de la salud: revisión sistemática 2024. *Mendeley Data, V1*, 10.17632/5nw8y7v3jt.1

[13] 13. Jaen-Varas D, Mari JJ, Asevedo E, Borschmann R, Diniz E, Ziebold C, et al. The association between adolescent suicide rates and socioeconomic indicators in Brazil: a 10-year retrospective ecological study. *Braz J Psychiatry.* 2019;41(5):389–95. 10.1590/1516-4446-2018-0223 PMC6796813 30785539

[14] 14. Machado DB, Rasella D, Dos Santos DN. Impact of Income Inequality and Other Social Determinants on Suicide Rate in Brazil. *PLoS one.* 2015;10(4):e0124934. 10.1371/journal.pone.0124934 PMC4416030 25928359

[15] 15. Dantas AP, de Azevedo UN de, Nunes AD, Amador AE, Marques MV. Analysis of suicide mortality in Brazil: spatial distribution and socioeconomic context. *Braz J Psychiatry*. 2018;40(1):12-18. 10.1590/1516-4446-2017-2241 PMC6899420 28832751

[16] 16. Luna Contreras M, Dávila Cervantes CA. Adolescentes en riesgo: factores asociados con el intento de suicidio en México. *Revista Gerencia y Políticas de Salud *2018;17(34):1-12. 10.11144/Javeriana.rgsp17-34.arfa

[17] 17. Rivera-Rivera L, Fonseca-Pedrero E, Séris-Martínez M, Vázquez-Salas A, Reynales-Shigematsu LM. Prevalencia y factores psicológicos asociados con conducta suicida en adolescentes. Ensanut 2018-19. *Salud Publica Mex*. 2020;62(6):672-681. 10.21149/11555 33620964

[18] 18. Manríquez Garcia N, Lara López F, Castro Lugo D. El suicidio y sus determinantes socioeconómicos en México: un análisis de panel de datos para las entidades federativas, 2010-2015. En: Universidad Michoacana de San Nicolás de Hidalgo. Modelos econométricos y sus aplicaciones en las ciencias sociales. 1 ed. México. 2020. p. 65-91. https://www.researchgate.net/publication/349442993_El_suicidio_y_sus_determinantes_socioeconomicos_en_Mexico_un_analisis_de_panel_de_datos_para_las_entidades_federativas_2010-2015

[19] 19. Murillo Gutiérrez LC, Quemba Mesa MP, Vargas Rodríguez LY, Florez Escobar IC, Contreras Briceño JI. Epidemiological behavior of suicide attempt in Colombian adolescents years 2016-2019: An ecological study. *Rev Latino-Am Enfermagem*. 2022;30:e3807. 10.1590/1518-8345.6240.3807 PMC9695283 36449926

[20] 20. Campo, A., Herazo, E. Pobreza, desigualdad y tasa de suicidio en Colombia, 2012. Duazary. 2014; 11(2), 126-130. Citado: 02/12/2024 Disponible en: https://www.redalyc.org/pdf/5121/512156302007.pdf

[21] 21. Campo-Arias A, Herazo E. Asociación entre desigualdad y tasa de suicidio en Colombia, 2012. *Duazary.* 2014;11(2):126-130 10.1016/j.rcp.2014.09.006

[22] 22. Gerstner RMF, Soriano I, Sanhueza A, Caffe S, Kestel D. Epidemiología del suicidio de adolescentes y jóvenes en Ecuador.* Rev Pan Am Salud Publica.* 2018;42:e100. 10.26633/RPSP.2018.100 PMC6385964 31093128

[23] 23. Mújica OJ. Cuatro cuestiones axiológicas de la epidemiología social para el monitoreo de la desigualdad en salud. *Rev Panam Salud Publica*. 2015;38(6):433-41. https://www.scielosp.org/pdf/rpsp/2015.v38n6/433-441/es 27440090

[24] 24. Organización Panamericana de la Salud. Determinantes e Inequidades en Salud. En Salud en las Américas. 2012; (12–59). Consulta: Diciembre 02, 2024. Disponible en: https://www.paho.org/es/documentos/determinantes-sociales-salud-region-americas-capitulo-salud-americas

[25] 25. Ordoñez Monak IA. Exploración de la Relación entre el Fenómeno del Suicidio en el Adulto Mayor y las Condiciones de Inequidad desde la Perspectiva de los Determinantes Sociales de la Salud Colombia:2009-2013. [Tesis de doctorado en Salud Pública]. Colombia, Universidad Nacional de Colombia; 2021. https://repositorio.unal.edu.co/handle/unal/80584

[26] 26. Morales-Borrero C, Borde E, Eslava-Castañeda J, Concha-Sánchez S. ¿Determinación social o determinantes sociales? Diferencias conceptuales e implicaciones praxiológicas. *Rev. salud pública.* 2013;15(6):797-808. https://www.scielosp.org/pdf/rsap/2013.v15n6/810-813/es 25124346

[27] 27. Naranjo Navas AD, Naranjo Navas CP. Homicidios y suicidios en relación con la inequidad en América Latina. *KAIRÓS*. 2022;5(9)9-27. 10.37135/kai.03.09.01

[28] 28. Durkheim E. El suicidio. Traducción Chaparro Sandra. Editorial Akal, S. A. 2012. Disponible en: https://circulosemiotico.wordpress.com/wp-content/uploads/2018/08/durkheim-c3a9mile-el-suicidio.pdf

[29] 29. Chandler A. Socioeconomic inequalities of suicide: Sociological and psychological intersections.* European Journal of Social Theory.* 2020;23(1),33–51. 10.1177/1368431018804154

[30] 30. Curtis B, Curtis C, Fleet R. Socio-economic factors and suicide: The importance of inequality. *New Zeland Sociology*. 2013;28(2):77-92. https://search.informit.org/doi/10.3316/informit.864982347297375

[31] 31. Harper S, Charters TJ, Strumpf EC, Galea S, Nandi A. Economic downturns and suicide mortality in the USA, 1980-2010: observational study. *Int J Epidemiol.* 2015;44(3):956-66. 10.1093/ije/dyv009 PMC4521126 26082407

[32] 32. Maclsaac, M, Bugeja, L, Jelinek, G. The association between exposure to interpersonal violence and suicide among women: a systematic review. *Aust N Z J Public Helath*. 2017;41(1):61-69. 10.1111/1753-6405.12594 27774704

[33] 33. Campo-Arias A, Suárez-Colorado YP, Caballero-Domínguez CC. Association between the use of Cannabis and elevated suicide risk in high school adolescents from Santa Marta, Colombia. *Biomedica.* 2020;40(3):569-577. 10.7705/biomedica.4988 PMC7666853 33030835

[34] 34. Dávila Cervantes CA, Luna Contreras M. Suicide attempt in teenagers: Associated factors. *Rev Chil Pediatr.* 2019;90(6):606-616. https://pubmed.ncbi.nlm.nih.gov/32186583/10.32641/rchped.v90i6.1012 32186583

[35] 35. Moreno G. Suicidio y depresión en adolescentes: una revisión de la literatura. Chilena de Salud Pública. 2019; 23(1): 31-41. Citado: 02/14/2024 Disponible en: https://pesquisa.bvsalud.org/portal/resource/en;/biblio-1371756

[36] 36. Soto-Sanz V, Ivorra-González D, Alonso J, Castellvi P, Rodríguez-Marín J, Piqueras JA. Revisión sistemática de programas de prevención del suicidio en adolescentes de población comunitaria. *Psicología clínica con niños y adolescentes.* 2019;6(3):62-75. https://dialnet.unirioja.es/servlet/articulo?codigo=7041029

[37] 37. Quispe A, Alvarez-Valdivia M, Loli-Guevara S. Metodologías Cuantitativas 2: Sesgo de confusión y cómo controlar un confusor. *Rev. cuerpo méd. HNAAA* 2020;13(2):205-12. 10.35434/rcmhnaaa.2020.132.67

